# A resource of lipidomics and metabolomics data from individuals with undiagnosed diseases

**DOI:** 10.1038/s41597-021-00894-y

**Published:** 2021-04-21

**Authors:** Jennifer E. Kyle, Kelly G. Stratton, Erika M. Zink, Young-Mo Kim, Kent J. Bloodsworth, Matthew E. Monroe, Undiagnosed Diseases Network, Carlos A. Bacino, Neil A. Hanchard, Richard A. Lewis, Jill A. Rosenfeld, Daryl A. Scott, Alyssa A. Tran, Patricia A. Ward, Lindsay C. Burrage, Gary D. Clark, Mercedes E. Alejandro, Jennifer E. Posey, Michael F. Wangler, Brendan H. Lee, William J. Craigen, Hugo J. Bellen, Sarah K. Nicholas, Bret L. Bostwick, Susan L. Samson, Alica M. Goldman, Paolo M. Moretti, Christine M. Eng, Donna M. Muzny, James P. Orengo, Tiphanie P. Vogel, Seema R. Lalani, David R. Murdock, Mahshid S. Azamian, Jordan S. Orange, Lisa T. Emrick, Shweta U. Dhar, Ashok Balasubramanyam, Lorraine Potocki, Shinya Yamamoto, Yaping Yang, Shan Chen, Fariha Jamal, Lefkothea Karaviti, Ronit Marom, Sharyn A. Lincoln, Chris A. Walsh, Alan H. Beggs, Lance H. Rodan, Joan M. Stoler, Gerard T. Berry, Laurel A. Cobban, Calum A. MacRae, Joel B. Krier, Edwin K. Silverman, Elizabeth L. Fieg, Richard L. Maas, Joseph Loscalzo, Aaron Aday, Susan Korrick, David B. Goldstein, Nicholas Stong, Jennifer A. Sullivan, Rebecca C. Spillmann, Loren D. M. Pena, Queenie K.-G. Tan, Nicole M. Walley, Yong-hui Jiang, Allyn McConkie-Rosell, Kelly Schoch, Vandana Shashi, Heidi Cope, Ingrid A. Holm, Isaac S. Kohane, Alexa T. McCray, Cecilia Esteves, Kimberly LeBlanc, Matthew Might, Emily Kelley, Elizabeth A. Worthey, Daniel C. Dorset, Braden E. Boone, Shawn E. Levy, Camille L. Birch, Angela L. Jones, Donna M. Brown, David P. Bick, J. Scott Newberry, Jozef Lazar, Thomas May, David A. Sweetser, Lauren C. Briere, J. Carl Pallais, Cynthia M. Cooper, Frances High, Melissa Walker, Heather A. Colley, Laura A. Mamounas, Teri A. Manolio, Elizabeth A. Burke, Rena A. Godfrey, Catherine A. Groden, William A. Gahl, Lynne A. Wolfe, Thomas C. Markello, C. Christopher Lau, David D. Draper, Sarah E. Gould, Michele E. Nehrebecky, Colleen E. Wahl, Gabriel F. Batzli, Ellen F. Macnamara, Jyoti G. Dayal, David J. Eckstein, John J. Mulvihill, Cynthia J. Tifft, Tiina K. Urv, Anastasia L. Wise, Jennifer L. Murphy, Andrea L. Gropman, Ellen M. Howerton, Donna M. Krasnewich, Jean M. Johnston, Barbara N. Pusey, David R. Adams, Valerie V. Maduro, May Christine V. Malicdan, Mariska Davids, Tyra Estwick, Donna Novacic, Prashant Sharma, Camilo Toro, Guoyun Yu, Babak Behnam, Precilla D’Souza, Carlos Ferreira, Marie Morimoto, Eva H. Baker, John Yang, Jean-Philippe F. Gourdine, Matthew Brush, Melissa Haendel, Euan A. Ashley, Jonathan A. Bernstein, Jacinda B. Sampson, Diane B. Zastrow, Noah D. Friedman, Jason D. Merker, Colleen E. McCormack, Paul G. Fisher, Jean M. Davidson, Annika M. Dries, Gregory M. Enns, Marta M. Majcherska, Chloe M. Reuter, Daryl M. Waggott, Jennefer N. Kohler, Terra R. Coakley, Kevin S. Smith, Matthew T. Wheeler, Devon Bonner, Liliana Fernandez, Jason Hom, Yong Huang, Shruti Marwaha, Chunli Zhao, Julian A. Martínez-Agosto, Esteban C. Dell’Angelica, Jeanette C. Papp, Emilie D. Douine, Stan F. Nelson, Martin G. Martin, Christina GS. Palmer, Neil H. Parker, Manish J. Butte, Amanda J. Yoon, Sandra K. Loo, Brent L. Fogel, Katrina M. Dipple, Janet S. Sinsheimer, Patrick Allard, Hayk Barseghyan, Naghmeh Dorrani, Hane Lee, Eric Vilain, Ascia Eskin, Genecee Renteria, Rebecca Signer, Jijun Wan, Allison Zheng, Monte Westerfield, John A. Phillips, Joy D. Cogan, John H. Newman, Amy K. Robertson, Rizwan Hamid, Anna Bican, Elly Brokamp, Laura Duncan, Mary Kozuira, Lynette Rives, Lisa Shakachite, Katrina M. Waters, Bobbie-Jo M. Webb-Robertson, David M. Koeller, Thomas O. Metz

**Affiliations:** 1 Biological Sciences Division, Earth and Biological Sciences Directorate, Pacific Northwest National Laboratory, Richland, WA 99352 USA; 2 Computing and Analytics Division, National Security Directorate, Pacific Northwest National Laboratory, Richland, WA 99352 USA; 3 Molecular and Medical Genetics, School of Medicine, Oregon Health and Science University, Portland, OR 97239 USA; 4 Baylor College of Medicine, Houston, Texas USA; 5 Boston Children’s Hospital, Boston, Massachusetts USA; 6 Brigham and Women’s Hospital, Boston, Massachusetts USA; 7 Columbia University, New York City, New York USA; 8 Duke University Medical Center, Durham, North Carolina USA; 9 Harvard Medical School, Boston, Massachusetts USA; 10 HudsonAlpha Institute for Biotechnology, Huntsville, Alabama USA; 11 Massachusetts General Hospital, Boston, Massachusetts USA; 12 National Institutes of Health, Bethesda, Maryland USA; 13 Oregon Health and Science University, Portland, Oregon USA; 14 Stanford University, Stanford, California USA; 15 University of California Los Angeles, Los Angeles, California USA; 16 University of Oregon, Eugene, Oregon USA; 17 Vanderbilt University Medical Center, Nashville, Tennessee USA

**Keywords:** Diseases, Mass spectrometry, Diagnostic markers, Metabolomics

## Abstract

Every year individuals experience symptoms that remain undiagnosed by healthcare providers. In the United States, these rare diseases are defined as a condition that affects fewer than 200,000 individuals. However, there are an estimated 7000 rare diseases, and there are an estimated 25–30 million Americans in total (7.6–9.2% of the population as of 2018) affected by such disorders. The NIH Common Fund Undiagnosed Diseases Network (UDN) seeks to provide diagnoses for individuals with undiagnosed disease. Mass spectrometry-based metabolomics and lipidomics analyses could advance the collective understanding of individual symptoms and advance diagnoses for individuals with heretofore undiagnosed disease. Here, we report the mass spectrometry-based metabolomics and lipidomics analyses of blood plasma, urine, and cerebrospinal fluid from 148 patients within the UDN and their families, as well as from a reference population of over 100 individuals with no known metabolic diseases. The raw and processed data are available to the research community so that they might be useful in the diagnoses of current or future patients suffering from undiagnosed disorders.

## Abstract (summary)

**Table:** 

Measurement(s)	Metabolomics • Lipidomics
Technology Type(s)	gas chromatography-mass spectrometry • Ultra High-performance Liquid Chromatography/Tandem Mass Spectrometry
Factor Type(s)	age group • sex
Sample Characteristic - Organism	Homo sapiens
Sample Characteristic - Environment	blood plasma material • urine material • cerebrospinal fluid material
Sample Characteristic - Location	United States of America

Machine-accessible metadata file describing the reported data: 10.6084/m9.figshare.13656581

## Background & Summary

Metabolites and lipids can be responsive to both genetic and environmental influences. Variations may occur due to host genes, disease states, lifestyle, diet, medications and the interaction with the gut microbiome^1^. Many rare diseases have genetic origins, but their symptoms can also be impacted by non-inherited causes such as infections, cancers, and other acquired conditions. Metabolomics and lipidomics analyses have been helpful in identifying inborn errors of metabolism, and in characterizing acquired metabolic conditions such as diabetes and metabolic syndrome^2,3^. These conditions are typically associated with a small number of metabolites and/or lipids that are significant outliers, and easily identified as abnormal.

In contrast, the metabolic changes in rare and undiagnosed diseases may be more subtle, consisting of complex patterns of minor changes of a large number of analytes rather than a few significant outliers. Due to the rare nature of these disorders, the number of individuals with a given phenotype is usually limited to one or just a few, precluding the use of the balanced study designs typically used in metabolomics. For these reasons the use of metabolomics and lipidomics analyses in the evaluation of rare and undiagnosed diseases presents many unique challenges.

The NIH Common Fund’s Undiagnosed Diseases Network (UDN) was established to accelerate the diagnosis and clinical management of rare or previously unrecognized diseases, and to advance research in disease mechanisms^4^. The UDN is composed of multiple clinical sites around the United States, and multiple research cores including DNA sequencing (whole exome and whole genome), model organisms (e.g., drosophila and zebrafish) and metabolomics^4,5^. As the Metabolomics Core for Phase I of the UDN, our role was to provide comprehensive untargeted measurements to identify qualitative and quantitative changes of metabolites (metabolomics) and lipids (lipidomics) in biofluids from probands (i.e. individuals with an undiagnosed disease accepted into the UDN) to assist in the evaluation and/or identification of the causes of rare and undiagnosed diseases. Here, we describe in detail the raw and processed metabolomics and lipidomics data from analyses of UDN patient samples and make the data available to the research community so that it might be useful in the diagnoses of current or future patients suffering from undiagnosed disorders. Our previous publication (Webb-Robertson *et al*.^6^) described the detailed statistical approach used for processing this same underlying data set, and so we refer readers to that work for more details on the statistical analyses employed.

## Methods

### Study design

**Fig. 1:**
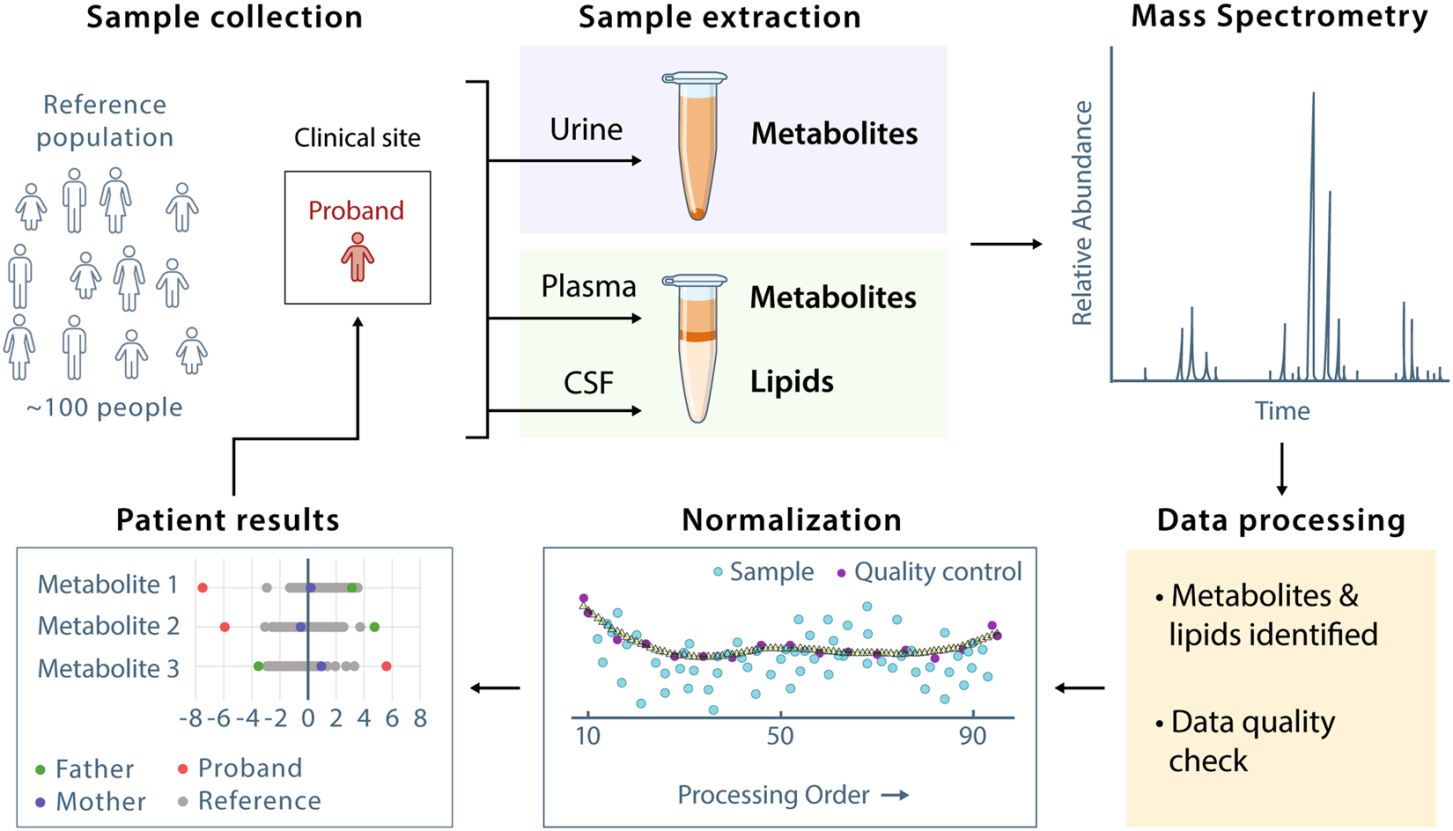
Overview of the study design. Biofluid samples were collected from probands at the UDN clinical sites and then extracted for metabolomics (urine, plasma, CSF) and lipidomics (plasma and CSF) analyses using chromatography coupled to mass spectrometry (GC-MS for metabolomics and LC-MS/MS for lipidomics). Data were pre-processed, including data quality checks, normalized, and compared against data from the reference population of healthy individuals. Metabolomics and lipidomics results in the form of Z-score, log2 fold change and p-value per metabolite and lipid of the proband (and associated family members, if applicable) were reported back to the respective UDN Clinical Site for diagnostic assistance.

The identification of metabolite and lipid outliers via metabolomics evaluation of individual probands by untargeted metabolomics required a normal or reference population for comparison. A reference dataset against which metabolomics data from UDN probands and their relatives could be compared was generated by metabolomics analysis of plasma, urine, and CSF from individuals with no known metabolic disease (Fig. 1). Approval for the study of the individuals in the UDN was provided by the National Institutes of Health under protocol number 15-HG-0130. The UDN is registered at ClinicalTrials.gov under identifier NCT02450851.

UDN probands suffer from undiagnosed diseases and thus are typically represented as a sample size of one; therefore, understanding normal variation within a proband’s condition is not possible. To address this issue, we performed power analyses of historical plasma and urine data from the Pacific Northwest National Laboratory (PNNL), assuming an uneven study design (e.g. n = 1 for probands and n = ≥(10–150) for healthy controls)^6^. This analysis determined that data from 80–120 healthy individuals would be required to perform a well-powered statistical analysis of the data from a UDN proband. This reference dataset is used to understand normal metabolome variation in a population of similar demographics to the UDN population, which is essential for evaluating the metabolome and lipidome data from individual UDN probands and characterizing the pathophysiology and etiology of their undiagnosed disease.

### Reference population

The composition of the reference population (approximately 50% children (<18 years of age) and approximately 50% female) was selected to represent the demographics of the participants enrolled in the Undiagnosed Diseases Program, an NIH intramural program upon which the UDN is based^7^. Biofluids for the reference population included samples collected from the Oregon Clinical & Translational Research Institute Biolibrary (adult plasma and urine), the Oregon Health & Science University Layton Aging and Alzheimer’s Research Center (adult CSF), the Vanderbilt University Metabolic Screening Laboratory (paediatric plasma), the Mayo Clinic Biochemical Genetics Laboratory (paediatric and young adult urine and CSF), and BioIVT (adult CSF)^6^ (Table 1**;** ***figshare***^8^ **(‘Demographic information for reference population’)**). The individuals composing the reference dataset also consented to sample collection under Institutional Review Boards (IRB) at the respective institutions.

**Table 1:** Demographics of reference population per biofluid type.

	Plasma (n = 136)	Urine (n = 102)	CSF (n = 149)
***Sex***			
Female	67 (49%)	55 (54%)	79 (53%)
Male	69 (51%)	47 (46%)	70 (47%)
***Age group (years)***			
0–0.5	0 (0%)	12 (12%)	16 (11%)
0.6–1.9	23 (17%)	4 (4%)	4 (3%)
2–10	53 (39%)	26 (25%)	25 (17%)
11–17	6 (4%)	8 (8%)	11 (7%)
18–30	23 (17%)	21 (21%)	4 (3%)
31–60	22 (16%)	22 (22%)	29 (19%)
>60	9 (7%)	9 (9%)	60 (40%)

For the paediatric and young adult CSF, due to the limited volumes available (100 µl), samples were pooled to reach the required volume of 200 µl for metabolomics analysis. Each CSF paediatric and adolescent reference sample is thus composed of two individuals of the same sex and similar age (e.g., 2 years old combined with 3 months old, and 14 years old combined with 16 years old).

### Biofluid collection for UDN participants and sample management

Biofluids were collected from UDN probands by the UDN clinical site at which the individual was evaluated. Written consent from all UDN participants and/or legal guardians was provided prior to sample collection and approved IRB. For each sample, the collection time, fasting state and duration, symptoms, diet supplements, and medications were documented. (***figshare***^8^ **(‘Listing of metabolomics and lipidomics raw data files’**). To assist in determination of potential genetic and environmental influences on the metabolomics findings, when it was possible samples were also collected from unaffected family members. Metabolomics and lipidomics analyses were conducted on 281 UDN participants, including 148 probands and 133 family members (101 unaffected, 25 affected, 7 unknown) (***figshare***^8^ **(‘Listing of metabolomics and lipidomics raw data files’**). This comprised 540 biofluid samples for analysis (295 plasma, 239 urine, and 6 CSF) (Table 2). Combining the reference population and the UDN participants, mass spectrometry analyses were conducted on 2781 biofluid samples. UDN probands with diagnoses are available (***figshare***^8^ **(‘UDN probands with available diagnoses’)**).

**Table 2:** Summary of mass spectrometry (MS) sample analyses per biofluid for the reference population and UDN cohort.

MS analysis	Plasma	Urine	CSF
***Reference***			
Metabolite (GC-MS)	136	102	149
Lipid (LC-MS/MS)	272	204	298
***UDN***			
Metabolite (GC-MS)	295	239	6
Lipid (LC-MS/MS)	590	478	12
***Total biofluid MS analyses***	***1293***	***1023***	***465***

Blood samples for plasma were collected in purple top EDTA Vacutainer® tubes. The blood was centrifuged at 10 000 × *g* for 10 minutes at 4 °C. Three 50 µl aliquots of plasma were transferred into 0.5 mL Sarstedt Biosphere® SC Micro Tubes. Samples were flash frozen in liquid nitrogen or quick frozen in dry ice/ethanol prior to storage in either a −80 °C or liquid nitrogen freezer with appropriate labels (ID, sample type, and collection date).

Urine samples were requested to be the first morning void and were collected in a polypropylene container. The urine was centrifuged at 1000 × *g* for 5 minutes at 4 °C to remove any cells and particulates. Three 100 µl aliquots were transferred into 0.5 mL Sarstedt Biosphere® SC Micro Tubes and flash frozen in liquid nitrogen or quick frozen in dry ice/ethanol prior to storage in either a −80 °C or liquid nitrogen freezer with appropriate labels (ID, sample type, and collection date).

CSF was collected by lumbar puncture in the L3/L4 or the L4/L5 inter-space. If the samples were not blood contaminated, the sample tubes were placed on ice (or dry ice if available), and then transferred to a −80 °C freezer. If the samples were blood contaminated, the samples were centrifuged immediately (prior to freezing) and the clear CSF transferred to new tubes. Three 200 µl aliquots were transferred into 0.5 mL Sarstedt Biosphere® SC Micro Tubes and flash frozen in liquid nitrogen or quick frozen in dry ice or ethanol prior to storage in a −80 °C freezer with appropriate labels (ID, sample type, and collection date)

All biofluid samples were shipped to the Pacific Northwest National Laboratory on dry ice and stored in −70 °C freezers until sample processing for mass spectrometry (MS) analysis.

### Quality control samples

The NIST SRM 1950 was used as a plasma QC^9,10^. The NIST QC is composed of 100 healthy individuals between 40–50 years old, an equal number of men and women, and a race distribution representative of the US population. The NIST QC is a commercially available reference material (certified until year 2023) and was chosen due to the multi-year nature of this study. For urine and CSF, as no commercially available reference materials were identified, pools were generated from the reference population for each respective biofluid and used as QCs.

### Sample batches

Sample batches were formed based on the number of analyses that could be performed in approximately one day (~33 analyses). Randomized run orders were generated based on sex, age, ethnicity (if provided), family association, and clinical site (if samples from more than 1 clinical site were available at the time of batching) (see Technical Validation section) prior to extraction, sample preparation, chemical derivatization of metabolites, and instrument analysis runs.

The instrument run order included a batch structure that enabled data normalization via Quality Control (QC)-based Robust Locally Estimated Scatterplot Smoothing (LOESS) Signal Correction (QC-RLSC)^11^ to specifically account for batches of samples that were not analysed back to back but dispersed over a longer timeframe^6^ (Table 3). The Pilot batches were the initial batches to be analysed and were used to confirm the normalization approach. Both Pilot and Project batches were processed using the same methodologies. This normalization method required a batch structure with specific placement of QC samples. For GC-MS analyses, the batch began with 2 blanks, 1 fatty acid methyl ester (FAME), 1 blank, 3 QCs, samples with evenly dispersed single QCs, and ending with 2 QCs. LC-MS/MS batches were similar except there was no FAME and a blank was run after the first 3 QCs, the middle QC, and at the very end to assess carryover^6^.

**Table 3:** Batch start date for the mass spectrometry analysis of the reference population and subsequent UDN participant samples per instrument type and biofluid.

Analysis Batch	Metabolomics (GC-MS)			Lipidomics (LC-MS/MS)	
	Plasma	Urine	CSF	Plasma	CSF
Reference_population	06-Jun-16	07-Dec-16	31-Jan-17	22-Sept-16	03-Mar-17
UDN_Pilot1	22-Jun-16	—	—	03-Oct-16	—
UDN_Pilot2	05-Oct-16	—	—	04-Oct-16	—
UDN_Project01	01-Dec-16	21-Dec-16	26-Sep-17	08-Dec-16	10-Oct-17
UDN_Project02	27-Feb-17	22-Dec-16	—	27-Mar-17	—
UDN_Project03	27-Sep-17	02-Oct-17	—	06-Oct-17	—
UDN_Project04	20-Sep-17	03-Oct-17	—	07-Oct-17	—
UDN_Project05	29-Sep-17	04-Oct-17	—	08-Oct-17	—
UDN_Project06	21-Sep-17	05-Oct-17	—	09-Oct-17	—
UDN_Project07	24-Oct-17	25-Oct-17	—	24-Oct-17	—
UDN_Project08	12-Dec-17	21-Feb-18	—	11-Dec-17	—
UDN_Project09	20-Feb-18	16-May-18	—	03-Feb-18	—
UDN_Project10	14-May-18	17-May-18	—	21-May-18	—
UDN_Project11	15-May-18	01-Oct-18	—	22-May-18	—
UDN_Project12	02-Oct-18	—	—	18-Oct-18	—

### Extraction of metabolites and lipids from plasma and CSF

For plasma and CSF, 50 μL and 200 μL, respectively, were used for metabolite and lipid extraction using a modified Folch extraction^12^, the MPLEx protocol^13^. Prior to extraction, samples were transferred to MμlTI SafeSeal Sorenson microcentrifuge tubes. To the plasma, 50 μL of GC-MS internal standards (malonic acid-d4, succinic acid-d4, glycine-d5, citric acid-d4, fructose 13C6, L-tryptophan-d5, lysine-d4, alanine-d7, stearic acid-d35, benzoic acid-d5, octanoic acid-d15 at a final concentration of 1 μg/μL each)^11^ and 10 μL of LC-MS internal standards (SPLASH™ Lipidomix® Mass Spec Standard, Avanti Polar Lipids, Inc.) were added. To the CSF, 50 μL of GC-MS internal standards (fructose 13C6, L-tryptophan-d5, lysine-d4, alanine-d7, stearic acid-d35, benzoic acid-d5, octanoic acid-d15 at a final concentration of 1 μg/μL each) and 10 μL of LC-internal standards ((PC(17:0/14:1) at 1 μg/μL, LPC(19:0) at 0.01 μg/μL, and TG(17:0/17:1/17:0)-d5 at 0.01 μg/μL) were added. Cold (−20 °C) chloroform/methanol (2:1, v/v) was added in a 4-fold excess to the sample volume. Samples were vortexed for 10 seconds to facilitate mixing of samples and solvent, allowed to sit on ice for 5 minutes, and then vortexed again for 10 seconds. Then, the samples were centrifuged to facilitate separation of a top hydrophilic layer containing polar metabolites and a bottom hydrophobic layer containing lipids. The hydrophilic layers were transferred into new 2.0 mL glass autosampler vials, evaporated to dryness *in vacuo*, and stored dry at −20 °C until chemical derivatization (see below). The lower hydrophobic layers containing the total lipid extract (TLE) were transferred into new 1.7 mL glass autosampler vials, evaporated to dryness *in vacuo*, and stored at −20 °C in 500 μL of chloroform/methanol (2:1, v/v) until instrument analysis.

### Extraction of metabolites from urine

For urine, 100 μL was used for metabolite extraction, as previously described^14^. Samples were transferred to MμlTI SafeSeal Sorenson microcentrifuge tubes to which 50 μL of GC-MS internal standards (malonic acid-d4, fructose 13C6, L-tryptophan-d5, lysine-d4, alanine-d7, stearic acid-d35, benzoic acid-d5, octanoic acid-d15 at a final concentration of 1 μg/μL each) and 100 μL of a 1 mg/mL solution of urease prepared in water were added. The samples were incubated for 30 minutes at 37 °C with mild shaking to deplete urea. Metabolites were then extracted with concomitant protein precipitation by addition of 1 mL of cold (−20 °C) methanol. Samples were vortexed for 30 seconds and precipitated proteins were isolated by centrifugation. The supernatants were transferred to glass autosampler vials and then dried *in vacuo*. Metabolite extracts were stored dry at −20 °C until chemical derivatization (see below).

### Chemical derivatization of metabolites

Polar metabolites were chemically derivatized prior to metabolomics analysis. Two post-extraction standards (pentadecanoic acid-d3 and 3-hydroxymyristic acid-d5 at 1 μg/μL final concentration) were added to monitor instrument performance. Chemical derivatization of metabolites was previously detailed^14^. To protect carbonyl groups and reduce the number of tautomeric isomers, 20 μL of methoxyamine in pyridine (30 mg/mL) was added to each sample, followed by vortexing for 30 seconds and incubation at 37 °C with generous shaking for 90 minutes. To derivatize hydroxyl and amine groups to trimethylsilylated (TMS) forms, 80 μL of N-methyl-N-(trimethylsilyl)trifluoroacetamide (MSTFA) with 1% trimethylchlorosilane (TMCS) was added to each vial, followed by vortexing for 10 seconds and incubation at 37 °C with shaking for 30 minutes. The samples were allowed to cool to room temperature and were analysed the same day.

### GC-MS analysis

An Agilent GC 7890 A coupled with a single quadrupole MSD 5975 C was used to analyze chemically derivatized metabolites. GC-MS analysis was previously detailed^14^. Briefly, 1 μL of each sample was injected onto a HP-5MS column (30 m × 0.25 mm × 0.25 μm; Agilent Technologies, Inc). The injection port temperature was held at 250 °C throughout the analysis. The GC oven was held at 60 °C for 1 minute after injection then increased to 325 °C by 10 °C/min, followed by a 5-minute hold at 325 °C. Total analysis time was 34 minutes per injection. The helium gas flow rates were determined by the Agilent Retention Time Locking function based on analysis of deuterated myristic acid. Data were collected over the mass range 50–550 m/z. A mixture of fatty acid methyl esters (C8-C28) was analysed once per day at the beginning of each batch together with the samples for retention index alignment purposes during subsequent data analysis.

### LC-MS analysis

Stored plasma TLEs were dried *in vacuo* (30 min) and reconstituted in 200 μL of methanol containing post-extraction internal standards (PE(17:0/14:1) and PI(17:0/14:1) at a final amount of 0.05 μg and 0.01 μg, respectively). Stored CSF TLEs were dried *in vacuo* and reconstituted in 50 μL of methanol containing the same post-extraction internal standards at a final amount of 0.02 μg each. The TLEs were analysed as outlined in Kyle *et al*.^15^. A Waters Acquity UPLC H class system interfaced with a Velos-ETD Orbitrap mass spectrometer was used for LC-ESI-MS/MS analyses. 10 μl of reconstituted sample was injected onto a Waters CSH column (3.0 mm × 150 mm x 1.7 μm particle size) and separated over a 34-minute gradient (mobile phase A: ACN/H2O (40:60) containing 10 mM ammonium acetate; mobile phase B: ACN/IPA (10:90) containing 10 mM ammonium acetate) at a flow rate of 250 μl/minute. Eluting lipids were introduced to the MS via electrospray ionization in both positive and negative modes, and lipids were fragmented using higher-energy collision dissociation (HCD) and collision-induced dissociation (CID).

### Metabolite identification and data processing

Metabolite identifications and data processing were conducted as previously detailed^14^. GC-MS raw data files were processed using the Metabolite Detector software, version 2.0.6 beta^16^. Retention indices (RI) of detected metabolites were calculated based on the analysis of the FAMEs mixture, followed by their chromatographic alignment across all analyses after deconvolution. Metabolites were identified by matching experimental spectra to an augmented version of the Agilent Fiehn Metabolomics Retention Time Locked (RTL) Library^17^, containing spectra and validated retention indices. All metabolite identifications were manually validated. The NIST 08 GC-MS library was also used to cross validate the spectral matching scores obtained using the Agilent library and to provide identifications for metabolites that were initially unidentified. The three most abundant fragment ions in the spectra of each identified metabolite were automatically determined by Metabolite Detector, and their summed abundances were integrated across the GC elution profile. A matrix of identified metabolites, unidentified metabolite features, and their corresponding abundances for each sample in the batch were exported for statistics.

Processing the data from the analyses of the reference population resulted in the identification of 81 plasma polar metabolites (across 16 super classes and 27 classes as categorized in the Human Metabolome Database^18,19^, 116 urine metabolites (across 17 super classes and 28 classes), and 82 CSF metabolites (across 14 super classes and 26 classes) (Table 4)

**Table 4:** Number of metabolites identified in the reference population biofluids by metabolite class.

HMDB Metabolite Class	Plasma	Urine	CSF
Alcohols and Polyols	2	1	2
Alkylamines	1	—	—
Amines	—	1	1
Amino Acids and Derivatives	19	25	15
Anhydrohexose	1	—	1
Benzenoids	—	9	—
Benzoic Acid and Derivatives	1	—	—
Carboxylic Acids and Derivatives	6	10	4
Cyclic Alcohols and Derivatives	—	1	—
Diazines	1	—	—
Disaccharides	2	6	1
Fatty Acids and Conjugates	4	1	5
Furans	—	1	—
Glycerolipids	2	—	—
Glycerophospholipids	—	1	1
Glycosyl Compounds	—	1	1
Hydroxy Acids and Derivatives	6	15	11
Imidazolidines	—	1	—
Imidazopyrimidines	2	3	—
Indoles and derivatives	1	—	—
Keto acids and derivatives	5	1	1
Keto-Acids and Derivatives	1	1	2
Lactams	1	1	1
Lactones	—	—	2
Monosaccharides	6	12	11
Non-metal Oxoanionic Compounds	1	1	1
Organic carbonic acids and derivatives	—	—	1
Organic nitrogen compound	1	1	1
Organic Phosphoric Acids and Derivatives	1	—	—
Organooxygen compound	—	—	1
Phenols	1	—	—
Phenylpropanoids and polyketides	—	1	—
Piperidines	—	—	1
Purine Nucleosides and Analogues	—	3	—
Purine Nucleotides	1	—	—
Pyridines and derivatives	2	3	1
Pyrimidines and pyrimidine derivatives	—	1	1
Steroids and Steroid Derivatives	1	—	—
Sugar Acids and Derivatives	3	6	4
Sugar alcohols	4	7	8
Tetrapyrroles and Derivatives	1	1	1
Unclassified	4	1	3
*Total*	*81*	*116*	*82*

### Lipid identification and data processing

LC-MS/MS lipidomics data were analyzed using LIQUID (Lipid Informed Quantitation and Identification)^15^. Analysis parameters included an initial precursor mass error tolerance of 20 ppm (i.e. ±10 ppm), and fragment mass error tolerances of 20 ppm (±10 ppm) and 500 ppm (±250 ppm) for HCD and CID MS/MS events, respectively. Confident identifications were selected by manually evaluating the MS/MS spectra for diagnostic and corresponding acyl chain fragments of the identified lipid. In addition, the precursor isotopic profile, extracted ion chromatogram, and mass measurement error along with the elution time were evaluated. For certain lipids, multiple LC peaks having nearly identical MS/MS spectra were observed, suggesting the presence of lipid stereoisomers. In these cases, the stereochemistry of the lipid isomers could not be completely determined based on the LC-MS/MS data alone, and so these isomers are annotated with “_A”, “_B” or “_C” at the end of the lipid name. Typically, the mass measurement error of confidently identified lipids was within ± 2.5 ppm. Given the time-consuming nature of manual validation of each identified lipid, a library of confident lipid identifications was generated from the reference dataset and select UDN participants (3 NIST QCs, 6 pooled plasma of reference population, 2 reference individuals, and 3 UDN participant). All LC-MS/MS data were aligned and gap-filled to this target database for feature identification using the identified lipid name, observed *m/z*, and the retention time using MZmine 2^20^ (see ***figshare***^8^ **(‘Parameters used for MZmine2 processing of lipidomics data’)**). Data from each ionization type were aligned and gap-filled separately. Aligned features were manually verified and peak apex intensity values were exported for statistical analysis. All subsequent batches were aligned to this library of confident lipid identifications.

To correct for batch retention time (RT) shifts for alignment to the reference library, an in-house tool to correct for linear RT shifts was used. The instrument files were converted into.mzXML files using MSConvert^21^. Each file was associated with a target list containing the name, RT, and *m/z* of the internal standards within a batch and was imported into MZmine. As the internal standards alone did not elute across the entire gradient, two lipids that were present in all samples in positive mode (carnitine(10:1) and CE(18:1)) and one in negative mode (HexCer(d18:1/24:0) were included in the target list as they eluted near the start and end of the gradient. The peak alignment of each target was manually validated, and corrected if needed, and the RT of each target lipid was exported. These targets acted as anchor points for the RT correction. Using the RT anchors for each target, all instrument files within a batch were shifted and aligned to the reference and new.mzXML were generated for subsequent alignment in MZmine. For all batches aligned to the reference list, lipid identifications were randomly selected (approximately 30 lipids) and verified using LIQUID to ensure that identification in the reference and sample batches matched.

Processing the data from the analyses of the reference population resulted in the identification of 462 plasma lipids across 6 lipid categories and 23 lipid subclasses (as categorized by LipidMaps)^22–24^, and 208 CSF lipids across 6 lipid categories and 17 lipid subclasses (Table 5).

**Table 5:** Number of lipids identified in the reference population biofluids, by lipid subclass.

LipidMaps Lipid Subclass	Plasma	CSF
Carnitine Esters	4	—
NAE	2	1
CE	6	4
Ubiquinone	1	—
Cer	15	1
SM	35	26
HexCer	5	5
Hex2Cer	1	—
LPC	23	16
PC	88	52
LPCO	2	—
PCO	18	11
PCP	13	9
LPE	7	3
PE	26	13
PEO	6	4
PEP	40	9
LPI	2	—
PI	24	8
PS	1	—
DG	6	7
TG	137	39
*Total*	462	208

NAE = N-acylethanalamine; Cer = ceramide; SM = sphingomyelin; HexCer = Hexosylceramide; LacCer = Dihexosylceramide; LPC = monoacylglycerophosphocholine; PC = diacylglycerophosphocholine; LPCO = Monoalkylglycerophosphocholine; PCO = alkyl, acylglycerophosphocholine; PCP = 1Z-alkenyl acylglycerophosphocholine LPE = monoacylglycerophosphoethanolmine; PE = diacylglycerophosphoethanolmine; PEO = alkyl acylglycerophospho-ethanolamine; PEP = plasmalogen PE; LPI = PI = monoacylglycerophosphoinositol; PI = diacylglycerophosphoinositol; PS = diacylglycerophosphoserine; DG = diacylglyceride; TG = triacylglyceride.

### Statistical analysis

We have previously described in detail the statistical approach used for processing the data^6^, and briefly summarize this below. To facilitate the identification of potentially disease-associated analyte profiles of UDN participants, a reference population of individuals with no known metabolic diseases was established as described above. Batches of samples from UDN participants were analysed and compared to this reference population as outlined in Webb-Robertson *et al*.^6^. Briefly, quality control (QC) processing of the reference dataset includes log2 transformation and the removal of any identified or unidentified features not present in at least 10% of the samples. Samples with missing or low abundance values and an uncorrelated pattern of expression by Pearson correlation and rMd-PAV^25^ were assessed to determine whether the seemingly poor behaviour was most likely due to biological or to technical/sample preparation issues. If biological issues appeared to be the cause, the sample was retained in the current batch for further analysis; if technical issues appeared to be the cause, the sample was omitted from further analyses. QC processing for the participant samples included the same steps as for the reference samples; however, participant samples required stronger evidence before removal than reference samples.

Normalization of the reference data and the participant data was performed in two steps^6^. First, QC-RLSC accounted for batch effects, and was performed on a per-batch basis^11^. This required identical QC samples to be run in every batch of samples (for reference samples and UDN samples alike), as described above. Quality control–based robust LOESS signal correction (QC-RLSC) was implemented using the parameter values described previously^11^. Namely, a missingness threshold requiring the observation of a molecule in at least half of the QC samples, filtering of molecules with RSD above 30 percent, and possible polynomial degrees of first and second order. To account for differences in the amount of sample material analysed by GC-MS or LC-MS, QC-RLSC was followed by global median centering of each sample, where each log2 biomolecule value within a sample was normalized via subtraction of the corresponding sample median (also on the log2 scale)..

To identify unique features in the analyte profiles of participants, results were compared to those from the reference dataset^6^. A univariate approach was applied that compared the feature values of the participants to the mean and standard deviation of the feature values in the reference dataset using z-scores^26^. An absolute value z-score threshold was used to obtain a list of metabolites and/or lipids with outlying z-scores that may have potential diagnostic significance. Additionally, for a given participant and biomolecule, log2 fold changes relative to the reference data were computed as the difference between the participant’s log2 value and the median log2 value of the reference population.

## Data Records

The raw LC-MS and GC-MS data files in .raw and.D format, and converted files in .mzML format, and.CDF format, respectively were deposited and are publicly available at the MassIVE repository (MSV000084717^27^, MSV000085506^28^, MSV000085508^29^). The normalized values for all identified lipids and metabolites for the UDN individuals and reference population are also available in MassIVE. The evidence supporting the molecular identifications (e.g. fragment ion *m/z*, retention times) are provided (**figshare**^8^ **(‘The evidence supporting the molecular identifications (e.g. fragment ion m/z, retention times)’)**). The deposited data also contains the post-processed data including the log2 fold change, Z-score, and p-value for each lipid and metabolite per UDN individual in .csv format. For the lipid results, the identifications made in positive and negative ionization mode were consolidated into a single file. Family member data is included in the associated proband files. In addition, for the UDN probands that have been diagnosed, the diagnosis name and relevant gene information are provided (**figshare**^8^ **(‘UDN probands with available diagnoses’)**).

The data deposited to MassIVE contains up to three directories: peak, quant, and raw. Each biofluid data repository also contains automatically generated subdirectories prefixed with “ccms”. Users of the data should obtain data from the peak, quant, and raw directories listed above and detailed below:

peak/ → Peak_List_Files/

1. Reference_pop_(biofluid)_lipid/ = LC-MS/MS instrument files in .mzXML format for the reference population
2. Reference_pop_(biofluid)_metab/ = GC-MS instrument files in .cdf format for the reference population
3. UDN_(biofluid)_lipid/ = LC-MS/MS instrument files in .mzXML format for the UDN participant
4. UDN_(biofluid)_metab/ = GC-MS instrument files in .cdf format for the UDN participant

quant/ → Quantification_Results/

1. UDN_(biofluid)_lipid_normalized_data/ = normalized data files for the associated biofluid per lipidomics batch analysis for lipids identified in both positive (POS) and negative (NEG) ionization mode
2. UDN_(biofluid)_lipid_results/ = The lipidomics result file containing the log2 fold change, Zscore, and p-value for each identified lipid per UND participant (and relatives, if applicable)
3. UDN_(biofluid)_metab_normalized_data/ = normalized data files for the associated the biofluid per metabolomics batch analysis for the identified metabolites
4. UDN_(biofluid)_metab_results/ = The metabolomics result file containing the log2 fold change, Zscore, and p-value for each identified metabolite per UND participant (and relatives, if applicable)

raw/ → Raw_Spectrum_Files/

1. Reference_pop_(biofluid)_lipid/ = LC-MS/MS Thermo instrument files in .raw format for the reference population
2. Reference_pop_(biofluid)_metab/ = GC-MS Agilent instrument files in .D format for the reference population
3. UDN_(biofluid)_lipid/ = LC-MS/MS Thermo instrument files in .raw format for the UDN participant
4. UDN_(biofluid)_metab/ = GC-MS Agilent instrument files in .D format for the UDN participant

updates/ → 2020-03-10_alchemistmatt_b439c281 → quant → Quantification_Results →

UDN_urine_metab_normalized_data/ = normalized data file for the reference population for urine metabolites.

## Technical Validation

To ensure unbiased data production, randomization orders were created and followed for sample extraction, GC-MS derivatization, and MS run orders. Family units, meaning probands and their relatives, were analysed within the same batch. Batch sizes were limited to the number of samples that could be analysed by GC-MS in approximately one day due to the stability of the chemically derivatized metabolites. Approximately 33 samples composed a batch. Samples were randomized based on sex, age, ethnicity (if provided), family association, and clinical site (if samples from more than 1 clinical site were available at the time of batching). Randomization orders were created as sufficient samples accumulated to make up a new batch over the 2.5 years of the study. To monitor data quality, the QC samples (across all molecules) were evaluated with prior batches collected to verify removal of batch effects via normalization. In addition, on a batch-by-batch basis, data quality was monitored by visual inspection of the log2 internal standard values across all samples within a batch.

To evaluate the consistency of the data collection process the coefficient of variation (CV) was utilized. The CV is defined as the standard deviation divided by the mean and lower values signify lower variability. Using data from the reference population, QC samples per platform and biofluid, the median CV along with the first and third quartile are shown in Table 6. The median CV of the lipid negative mode CSF is the greatest, possibility due to the lower number of samples available and lipids identified.

**Table 6:** The coefficient of variation (CV) per platform and biofluid for the reference population QC samples calculated from raw values.

Platform & Biofluid	Min.	1st Qu.	Median	Mean	3rd Qu.	Max
Metabolite Urine	12.61	16.4	18.22	23.52	22.38	195.9
Metabolite Plasma	16.93	24.61	30.38	41.23	44.17	189.6
Metabolite CSF	12.68	23.53	30.27	41.89	49.80	201.4
Lipid POS Plasma	9.73	13.32	14.91	17.56	16.91	179.3
Lipid NEG Plasma	20.64	24.20	27.68	30.84	32.36	96.25
Lipid POS CSF	13.62	24.32	26.16	33.05	32.00	103.30
Lipid NEG CSF	25.16	32.08	46.34	45.16	51.22	87.42

Min = minimum CV,

1st Qu. = first quartile,

3rd Qu. = third quartile,

Max. = maximum CV,

POS = positive mode ionization,

NEG = negative mode ionization.

**Fig. 2:**
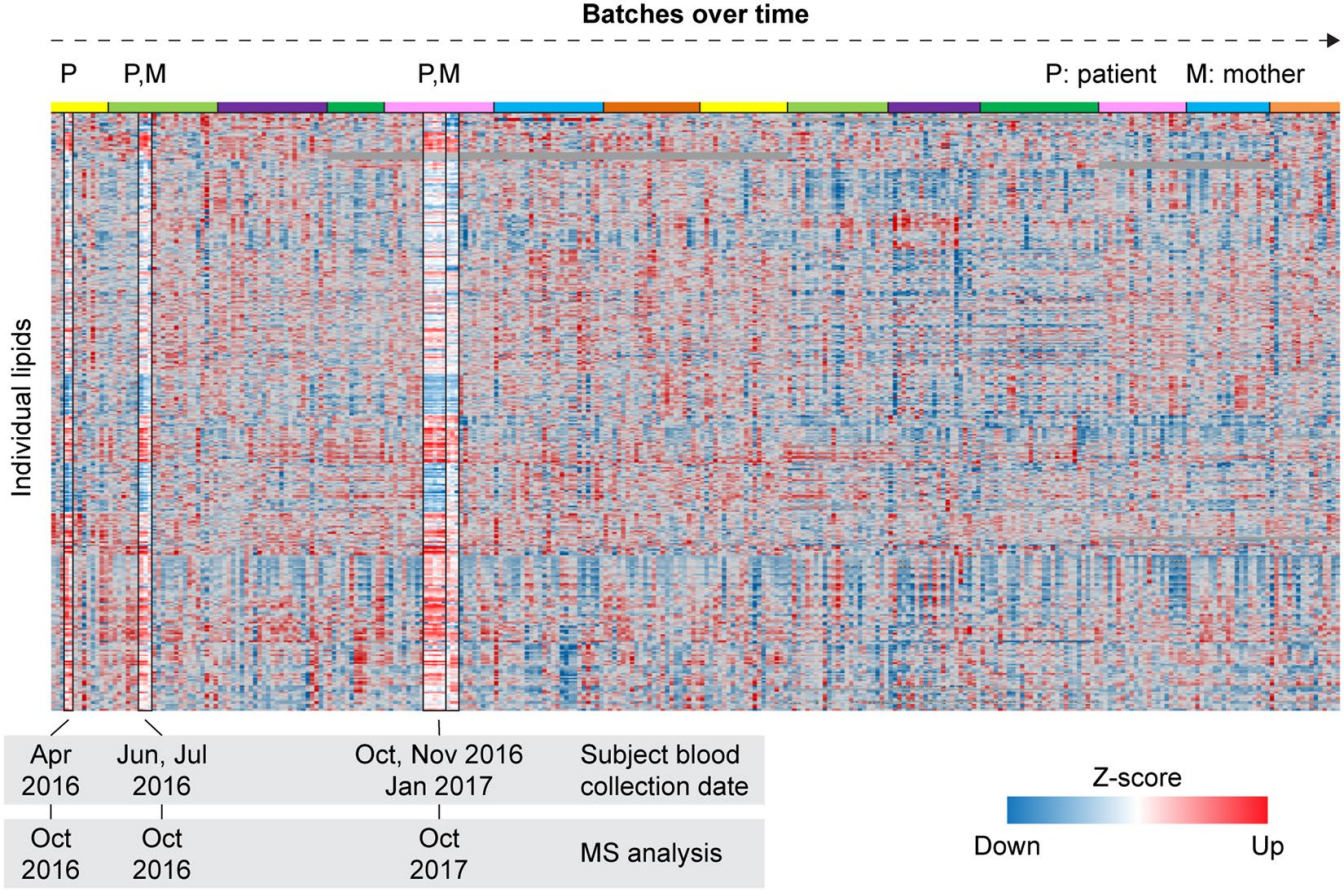
Z-score map of plasma lipidomics data for all UDN individuals (n = 294). One proband (P) and her mother (M) had samples analysed in multiple batches over the course of one year. The proband’s samples were collected at different times for each batch and analysed on the MS in October 2016 (2 batches, 2 samples per batch) and October 2017 (1 batch, 5 samples). The proband’s lipid profile remained very similar between each analysis batch. Top coloured bar indicates the different batches over time.

To evaluate the reproducibility of the results, we assessed the lipidomics results from one UDN proband from whom we had 9 samples collected over a period of 9 months. The samples were analysed in 3 different batches, separated by up to 1 year (Fig. 2). The proband’s mother had 3 samples analysed in two batches at time intervals coinciding with the proband’s samples. As shown in Fig. 2, the Z-score pattern of both the proband’s and the mother’s samples remain consistent between batches across the one-year timespan between the collection and analysis of the first and last set of samples (October 2016 to October 2017).

## Data Availability

Statistical processing and analyses were performed in R version 3.4.0. Quality control and median normalization were performed using the R package *pmartR* version 0.9.0, freely available on GitHub (https://github.com/pmartR/pmartR)^30^. Default parameter values for *pmartR* function calls were used. QC-RLSC and the calculation of log2 fold changes and z-scores were carried out using in-house R functions and are available on Github (https://github.com/pmartR/qcrlsc).

## References

[1] Nicholson JK (2012). Metabolic phenotyping in clinical and surgical environments. Nature.

[2] Garcia-Cazorla A, Mochel F, Lamari F, Saudubray JM (2015). The clinical spectrum of inherited diseases involved in the synthesis and remodeling of complex lipids. A tentative overview. Journal of inherited metabolic disease.

[3] Lamari F, Mochel F, Saudubray JM (2015). An overview of inborn errors of complex lipid biosynthesis and remodelling. Journal of inherited metabolic disease.

[4] Ramoni RB (2017). The Undiagnosed Diseases Network: Accelerating Discovery about Health and Disease. American journal of human genetics.

[5] Network, T. U. D. Undiagnosed Diseases Network. (2017).

[6] Webb-Robertson, B. M. *et al*. Statistically-driven Metabolite and Lipid Profiling of Patients from the Undiagnosed Diseases Network. Analytical chemistry, 10.1021/acs.analchem.9b03522 (2019). 10.1021/acs.analchem.9b03522 PMC7183858 31742994

[7] Gahl WA (2012). The National Institutes of Health Undiagnosed Diseases Program: insights into rare diseases. Genetics in medicine: official journal of the American College of Medical Genetics.

[8] Kyle JE (2021). figshare.

[9] Bowden JA (2017). Harmonizing lipidomics: NIST interlaboratory comparison exercise for lipidomics using SRM 1950-Metabolites in Frozen Human Plasma. Journal of lipid research.

[10] Simon-Manso Y (2013). Metabolite profiling of a NIST Standard Reference Material for human plasma (SRM 1950): GC-MS, LC-MS, NMR, and clinical laboratory analyses, libraries, and web-based resources. Analytical chemistry.

[11] Dunn WB (2011). Procedures for large-scale metabolic profiling of serum and plasma using gas chromatography and liquid chromatography coupled to mass spectrometry. Nature protocols.

[12] Folch J, Lees M, Sloane Stanley GH (1957). A simple method for the isolation and purification of total lipides from animal tissues. The Journal of biological chemistry.

[13] Nakayasu, E. S. *et al*. MPLEx: a Robust and Universal Protocol for Single-Sample Integrative Proteomic, Metabolomic, and Lipidomic Analyses. mSystems 1, 10.1128/mSystems.00043-16 (2016). 10.1128/mSystems.00043-16 PMC5069757 27822525

[14] Webb-Robertson BJ (2014). A Statistical Analysis of the Effects of Urease Pre-treatment on the Measurement of the Urinary Metabolome by Gas Chromatography-Mass Spectrometry. Metabolomics: Official journal of the Metabolomic Society.

[15] Kyle JE (2017). LIQUID: an-open source software for identifying lipids in LC-MS/MS-based lipidomics data. Bioinformatics (Oxford, England).

[16] Hiller K (2009). MetaboliteDetector: comprehensive analysis tool for targeted and nontargeted GC/MS based metabolome analysis. Analytical chemistry.

[17] Kind T (2009). FiehnLib: mass spectral and retention index libraries for metabolomics based on quadrupole and time-of-flight gas chromatography/mass spectrometry. Analytical chemistry.

[18] Djoumbou Feunang Y (2016). ClassyFire: automated chemical classification with a comprehensive, computable taxonomy. Journal of cheminformatics.

[19] Wishart DS (2013). HMDB 3.0–The Human Metabolome Database in 2013. Nucleic acids research.

[20] Pluskal T, Castillo S, Villar-Briones A, Oresic M (2010). MZmine 2: modular framework for processing, visualizing, and analyzing mass spectrometry-based molecular profile data. BMC bioinformatics.

[21] Adusumilli R, Mallick P (2017). Data Conversion with ProteoWizard msConvert. Methods in molecular biology (Clifton, N.J.).

[22] Fahy E (2005). A comprehensive classification system for lipids. Journal of lipid research.

[23] Fahy E (2009). Update of the LIPID MAPS comprehensive classification system for lipids. Journal of lipid research.

[24] Liebisch G (2013). Shorthand notation for lipid structures derived from mass spectrometry. Journal of lipid research.

[25] Matzke MM (2011). Improved quality control processing of peptide-centric LC-MS proteomics data. Bioinformatics (Oxford, England).

[26] Miller MJ (2015). Untargeted metabolomic analysis for the clinical screening of inborn errors of metabolism. Journal of inherited metabolic disease.

[27] Kyle JE (2020). MassIVE.

[28] Kyle JE (2020). MassIVE.

[29] Kyle JE (2020). MassIVE.

[30] Stratton KG, Webb-Robertson BM (2019). pmartR: Quality Control and Statistics for Mass Spectrometry-Based Biological Data..

